# Cytogenetic studies in four cultivated *Amaranthus* (Amaranthaceae) species

**DOI:** 10.3897/CompCytogen.v7i1.4276

**Published:** 2013-03-26

**Authors:** Marisa Graciela Bonasora, Lidia Poggio, Eduardo José Greizerstein

**Affiliations:** 1 Cátedra de Botánica Agrícola, Facultad de Agronomía, Universidad de Buenos Aires. Av. San Martín 4453 (C1417DSE), Buenos Aires, Argentina; 2 Laboratorio de Citogenética y Evolución, Departamento de Ecología, Genética y Evolución, Facultad de Ciencias Exactas y Naturales, Universidad de Buenos Aires. Intendente Güiraldes 2160 (C1428EGA), Buenos Aires, Argentina; 3 Cátedra de Mejoramiento Genético, Facultad de Ciencias Agrarias, UNLZ. Ruta 4 - Km. 2 - Llavallol (CP 1836), Argentina; 4 CONICET, Argentina

**Keywords:** *Amaranthus*, constitutive heterochromatin, NORs, FISH, DAPI-CMA_3_

## Abstract

In the present study, the chromosomes numbers were confirmed, 2n = 34 for *Amaranthus cruentus* Linnaeus, 1759, and 2n = 32 for *Amaranthus hypochondriacus* Linnaeus, 1753, *Amaranthus mantegazzianus* Passer, 1864, and *Amaranthus caudatus* Linnaeus, 1753. The distribution and variability of constitutive heterochromatin were detailed using DAPI-CMA_3_ banding technique. The position of the nucleolus organizer region (NOR) was observed using Ag-NOR banding (active loci) and fluorescent *in situ* hybridization (rDNA-FISH) in the four *Amaranthus* species. Variations in the amount of constitutive heterochromatin were detected both within the species and between them, with DAPI-CMA_3_ stain. One chromosome pair having a NOR was found in each studied accession, with exception of *Amaranthus caudatus* cv. EEA INTA Anguil. This accession presented four rDNA loci (FISH), being active two of them (Ag- banding).

## Introduction

The genus *Amaranthus* Linnaeus,1753 comprises about 50 herbaceous species, most of them are annuals. They grow preferentially in warm regions of America. Several species are cultivated as ornamentals (*Amaranthus caudatus* Linnaeus,1753), vegetables (*Amaranthus spinosus* Linnaeus, 1753 and *Amaranthus tricolor* Linnaeus, 1753), pseudocereals (*Amaranthus cruentus* Linnaeus, 1759, *Amaranthus hypochondriacus* Linnaeus, 1753, *Amaranthus mantegazzianus* Passer, 1864, and *Amaranthus caudatus* Linnaeus, 1753), and some of them are weeds.

Karyotypical studies in the genus are scarce, probably due to the small size of the chromosomes, which makes morphological analysis difficult (Grant 1959a, b, c). Updated data have indicated that there are two basic chromosome numbers, x = 16 and x = 17, and, in some cases, both numbers were cited for the same species (Grant 1959a, Pal and Khoshoo 1972, Pal 1972, 1973a, b, Pal et al. 1982, Poggio 1988). Pal et al. (1982) suggested that the gametic number n = 17originates from n = 16 through primary trisomy. Greizerstein and Poggio (1992) supported this hypothesis through the analysis of meiotic behavior of species and interspecific hybrids.

Studies carried out on chromosome morphology of some species of the genus have indicated variation in number of chromosome pairs with satellites. Palomino and Rubí (1991) reported the karyotypic formula in some cultivars of *Amaranthus hypochondriacus* and *Amaranthus cruentus*, suggesting the existence of six to ten pairs of chromosomes with satellites in different cultivars. Greizerstein and Poggio (1994) proposed karyotypic formulae of various accessions of cultivated species (*Amaranthus cruentus*, *Amaranthus hypochondriacus*, *Amaranthus mantegazzianus* and *Amaranthus caudatus*). In all studied species, only one pair of chromosomes with a satellite was found (Greizerstein and Poggio 1994). Kolano et al. (2001), indicated for two cultivars of *Amaranthus caudatus* the presence of one and two pairs of chromosomes with ribosomal hybridization signals using FISH technique with 45s ribosomal probes.

In the present work, the distribution and variability of constitutive heterochromatin and the number of active ribosomal organizer regions were studied in two different cultivars of the species *Amaranthus cruentus* (2n = 34), *Amaranthus mantegazzianus* (2n = 32), *Amaranthus hypochondriacus* (2n = 32), and *Amaranthus caudatus* (2n = 32). The aim of the study was to increase the knowledge about the genetic variability of the *Amaranthus* genus.

## Material and Methods

Analyses were performed in eight accessions of *Amaranthus*. Species cultivars and origin of the studied material are listed in Table 1.

**Table 1.** Species of *Amaranthus* analyzed with their respective cultivar.

**Species**	**Cultivar**	**Grant by:**
*Amaranthus cruentus* L.	Don Guiem, La Pampa.	Ing. Agr. Rosa M. de Troiani
*Amaranthus cruentus* L.	INDEAR SA	Dr. Francisco Trucco
*Amaranthus hypochondriacus* L.	Artaza	Ing. Agr. Rosa M. de Troiani
*Amaranthus hypochondriacus* L.	INDEAR SA	Dr. Francisco Trucco
*Amaranthus mantegazzianus* Passer.	Don Manuel	Ing. Agr. Rosa M. de Troiani
*Amaranthus mantegazzianus* Passer.	INDEAR SA	Dr. Francisco Trucco
*Amaranthus caudatus* L.	EEA INTA Anguil, La Pampa.	Ing. Agr. Guillermo Covas
*Amaranthus caudatus* L.	INDEAR SA	Dr. Francisco Trucco

Root tips from germinated seeds were pre-treated with colchicine at room temperature for 2 h, fixed in ethanol/glacial acetic acid 3:1 (v/v) for 24 hours and stored in ethanol 70% at -20 °C. Root tips were digested using a solution containing 2% cellulase and 20% pectinase (both w/v) for 90 min at 37 °C and dissected in 45% (v/v) aqueous acetic acid, squashed under a coverslip subsequently removed by freezing in dry ice, air dried and then stored in -20°C until use.

**Fluorochrome banding DAPI-CMA_3_:** Fluorochrome banding was performed in all cultivars according to Deumling and Greilhuber (1982). Slides were double stained with DAPI (4’-6-diamidino-2-phenylindole) and CMA_3_ (Chromomycin A_3_) and mounted in 1:1 (v/v) McIlvaine´s pH7 buffer-glycerol.

**FISH:** FISH was performed on mitotic cells according to Cuadrado and Jouve (1994) with minor modifications. The probe that was used is the pTa 71, that contains 9 kilobase (kb) *Eco*R1 repeat unit of 18S-5.8S-25S rDNA loci and spacers isolated from wheat, *Triticum aestivum* (Gerlach and Bedbrook 1979). Probe was labelled by nick translation with biotin 14- dUTP (Bionick Labelling System).

Slides were counterstained with 4’, 6-diamidino-2- phenylindole (DAPI) (1 μg McIlvaine’s citrate buffer/mL, pH = 7) for 10 min at room temperature, and subsequently mounted in antifade solution and examined with a Leica epifluorescence microscope with appropriate filters. Photographs were taken using a digital camera.

**Ag-staining:** Silver staining technique was carried out according with Neves et al. (1997).

## Results

**Figure 1.**
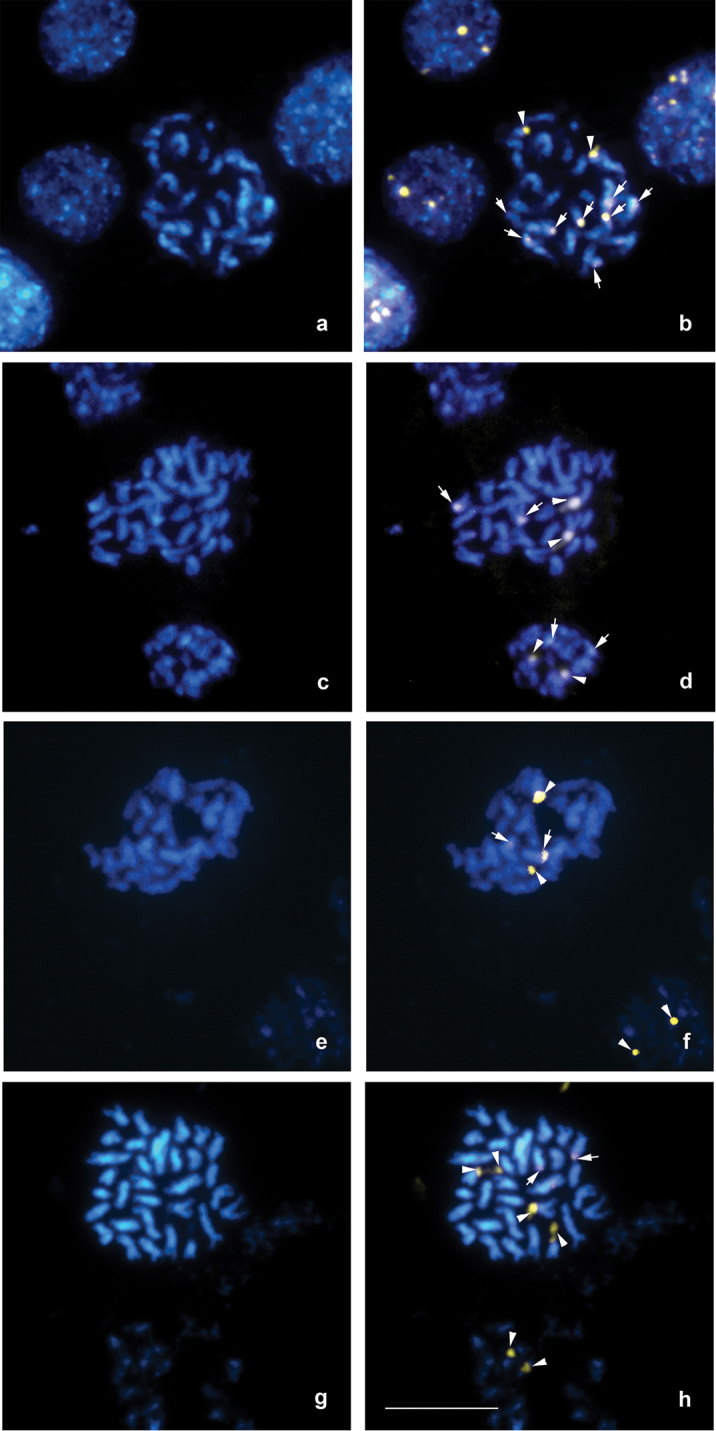
Metaphase cells with CMA_3_/DAPI banding **a** DAPI **b** CMA_3_ of *Amaranthus cruentus* cv. INDEAR **c** DAPI **d** CMA_3_ of *Amaranthus hypochondriacus* cv. Artaza **e** DAPI **f** CMA_3_ of *Amaranthus mantegazzianus* cv. INDEAR **g** DAPI and **h** CMA_3_) of *Amaranthus caudatus* cv. INDEAR. The arrow indicates CMA_3_+ band, and the arrowhead CMA_3_+/DAPI+ band. Bar = 5 μm.

Interstitial bands CMA_3_+/DAPI+ showed differences among cultivars (Fig. 1 and Table 2). The species *Amaranthus cruentus* cv. Don Guiem and cv. INDEAR showed two DAPI-/CMA_3_+ bands. Moreover, *Amaranthus cruentus* cv. INDEAR presented eight DAPI+/CMA_3_+ bands (Figs 1a and b).

**Table 2.** Variation in DAPI/CMA_3_ banding.

**Species**	**Cultivate**	**Band DAPI-/CMA_3_+**	**Band DAPI+/CMA_3_+**
*Amaranthus cruentus*	Don Guiem	2	-
*Amaranthus cruentus*	INDEAR SA	2	8
*Amaranthus hypochondriacus*	Artaza	2	2
*Amaranthus hypochondriacus*	INDEAR SA	2	6
*Amaranthus mantegazzianus*	Don Manuel	2	-
*Amaranthus mantegazzianus*	INDEAR SA	2	2
*Amaranthus caudatus*	EEA INTA Anguil	4	2
*Amaranthus caudatus*	INDEAR SA	4	2

The species *Amaranthus hypochondriacus* cv. Artaza showed two DAPI+/ CMA_3_+ bands, while the cv. INDEAR had six DAPI+/ CMA_3_+ bands (Figs 1c and d).

In *Amaranthus mantegazzianus* species cv. INDEAR showed two bands of DAPI-/CMA_3_+ and two DAPI+/CMA_3_+ bands, while in cv. Don Manuel only two DAPI-/CMA_3_+ bands were revealed (Figs 1e and f).

*Amaranthus caudatus* cv. EEA INTA Anguil and cv. INDEAR had four DAPI-/CMA_3_+ bands and two DAPI+/CMA_3_+ bands (Figs 1g and h).

**Figure 2.**
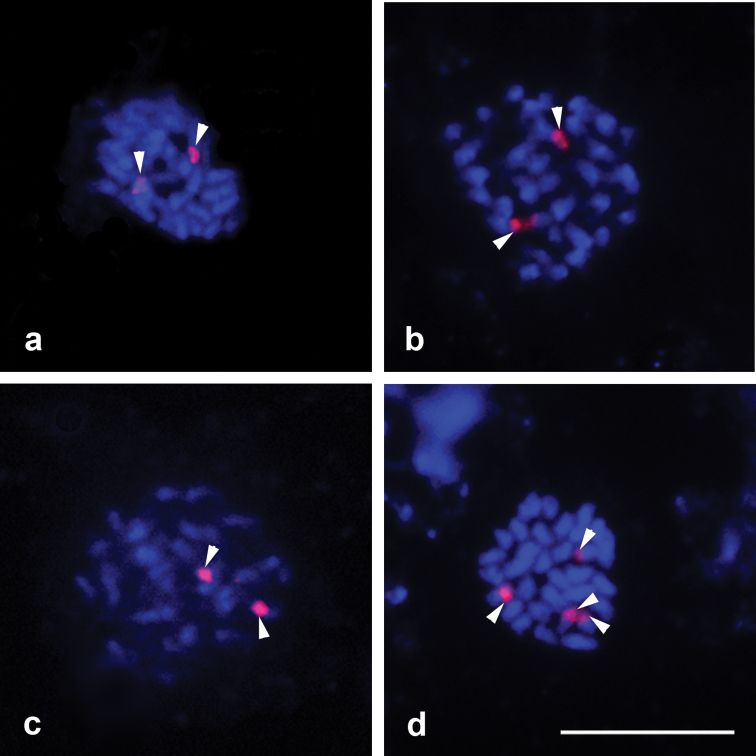
FISH with 18s ribosomal DNA **a** *Amaranthus cruentus* cv. Don Guiem **b** *Amaranthus hypochondriacus* cv. Artaza **c** *Amaranthus mantegazzianus* cv. Don Manuel **d** *Amaranthus caudatus* cv. EEA INTA Anguil the arrow indicates four signals. Bar = 5 μm.

FISH (Fig. 2) revealed two ribosomal hybridization signals for all studied cultivars except *Amaranthus caudatus* cv. EEA INTA Anguil (Fig. 2d) which presented four of them.

**Figure 3.**
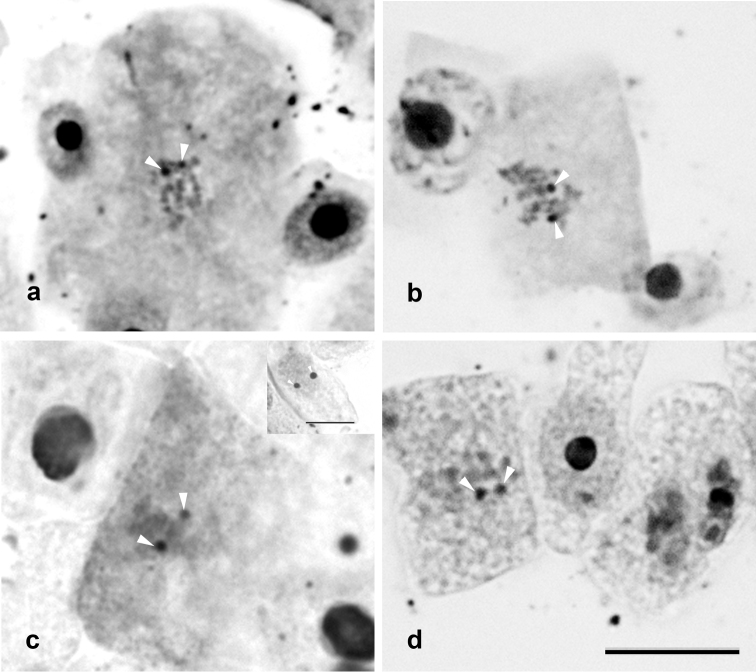
Ag-banding, **a** *Amaranthus cruentus* cv. INDEAR **b** *Amaranthus hypochondriacus* cv. Artaza **c** *Amaranthus mantegazzianus* cv. INDEAR and **d** *Amaranthus caudatus* cv. EEA INTA Anguil. Bar = 10 μm.

Ag-NOR technique (Fig. 3) allowed detection of one pair of chromosomes with active NOR in all studied materials. The results with FISH technique agree with the silver staining, which revealed two active NORs in the previous interphase. In the case of the four signals in *Amaranthus caudatus* cv. EEA INTA Anguil, only two would be coincident with active NORs.

## Discussion

All the studied accessions of *Amaranthus hypochondriacus*, *Amaranthus mantegazzianus* and *Amaranthus caudatus* presented the chromosome number 2n = 32and 2n = 34 for *Amaranthus cruentus*, which is in agreement with previous report (Greizerstein and Poggio 1994).

DAPI-CMA_3_ banding showed that all the species and their cultivars had DAPI+ intersticials bands. In all accessions, two DAPI-/CMA_3_+ bands were detected, which were coincident with active NOR sites, even in the species *Amaranthus caudatus* which exhibited four of them. Nevertheless, differences among cultivars in the same species respect the number of DAPI+/CMA_3_+ bands were found. For example, *Amaranthus cruentus* cv. INDEAR showed the higher number of these bands and *Amaranthus mantegazzianus* cv. Don Manuel did not present any. In the rest of the cultivars, two to six bands were observed, indicating the existence of inter and intraspecific differences in the quality and the amount of heterochromatin. Double staining with CMA_3_ and DAPI is the combination most used to differentiate chromosome bands and for NOR identification (Guerra 2000). Some studies demonstrated NOR sites coincident with DAPI-/CMA_3_+ bands (Guerra 2000; Barros e Silva and Guerra 2010). Due to CMA_3_ marks the presence of rich in CG sequences, this technique not only highlights the NORs sites, but also other regions in the genome (Guerra 2000). According to this statement, Kolano et al. (2001) found that the amount of CMA_3_+ bands in two other cultivars of *Amaranthus caudatus* could be more numerous than rDNA sites.

All studied cultivars of *Amaranthus cruentus*, *Amaranthus hypochondriacus* and *Amaranthus mantegazzianus* presented two hybridization signals by FISH. *Amaranthus caudatus* presented two hybridization signals in the cv. INDEAR, but four signals in cultivar EEA INTA Anguil. A similar result was detected, in the same species, by Kolano et al. (2001). They found one pair of signals in the cultivar Kiwicha 3, and two pairs in cv. Kiwicha Molinera, using FISH with ribosomal DNA probe. In the present work, four signals were detected in the cultivar EEA INTA Anguil, two of them were not coincident with Ag-NOR bands, which indicates the presence of inactive ribosomal loci.

In agreement with Greizerstein and Poggio (1994), we have detected a single pair of active NOR bands. The chromosomes carrying satellites, according these authors, were the fourth pair for *Amaranthus cruentus*, and the sixth pair for *Amaranthus hypochondriacus*, *Amaranthus mantegazzianus* and *Amaranthus caudatus*. These pairs of chromosomes could be the pair bearing the Ag-NOR bands, and they could be coincident with FISH results. However, Palomino and Rubí (1991) reported the presence of six to ten pairs of chromosomes with satellites. These differences could be showing a wide genetic variation masked by the few studied cultivars.

The difference in the amount of active NORs in the species *Amaranthus caudatus* cv. EEA INTA Anguil could be due to different sources. There are organisms which contain multiple NORs and many of them are silenced by epigenetic mechanisms. This silencing state of the NOR could be inherited by subsequent generations (McStay and Grummt 2008). The variation in the number of regions among cultivars could be due to this mechanism, or be a by-product of breeding programs.

To summarize, our results support the hypothesis that the cultivated *Amaranthus* species have two active NORs regions. Furthermore, the number of DAPI+/CMA_3_+ bands allowed the characterization and identification of heterochromatin in cultivars and species. However, it would be interesting to study others cultivars and native populations.
